# Smoking and gender modify the effect of *TWIST* on patient survival in head and neck squamous carcinoma

**DOI:** 10.18632/oncotarget.20682

**Published:** 2017-09-06

**Authors:** Yun Zhu, Wenjuan Zhang, Ping Wang

**Affiliations:** ^1^ Department of Otorhinolaryngology, Union Hospital, Tongji Medical College, Huazhong University of Science and Technology, Wuhan, 430022, China; ^2^ Department of Pediatrics, Union Hospital, Tongji Medical College, Huazhong University of Science and Technology, Wuhan, 430022, China; ^3^ Cancer Genetic Laboratory, Department of Molecular and Human Genetics, Baylor College of Medicine, Houston, TX, 77025, USA

**Keywords:** prognosis, twist-related protein, carcinoma, male, smoking

## Abstract

**Purpose:**

TWIST is a critical factor for predicting prognosis in several human cancers. Here, we study the prognostic significance of *TWIST1* and *TWIST2* in Head and Neck squamous cell carcinoma (HNSCC) as well as interactions of *TWISTs* with both gender and smoking in patient survival.

**Methods:**

upper quartile normalized RNA-seq V2 RSEM values of *TWIST1* and *TWIST2* expressions were retrieved from a TCGA HNSCC dataset. Kaplan-Meier survival curves were used to assess the associations of *TWIST1* and *TWIST2* with patient survival, and multivariate Cox proportional hazards regression models were used to estimate the hazards ratios (HRs) and their 95% confidence intervals (CIs).

**Results:**

Survival analyses showed that high *TWIST1* expression was associated with a poor overall survival at a borderline significance level, while a superior but not statistically significant overall survival was observed in high *TWIST2* expression. The multivariate Cox proportional hazards regression model showed a significantly elevated risk of death (HR=1.37, p = 0.038) in patients with high *TWIST1* compared to low *TWIST1,* and a borderline significantly decreased risk of death (HR = 0.74, p = 0.055) in patients with high *TWIST2* compared to low *TWIST2*. Further stratification analyses showed that increased risks of death were found significantly in male and borderline significantly in smoker patients with high *TWIST1* compared to low one, and a significantly decreased risk of death in non-smoker patients with high *TWIST2* compared to low one.

**Conclusions:**

*TWIST1* and *TWIST2* are differentially associated with HNSCC patient survival. Gender and smoking could modify the effect of *TWIST*s on the risk of death in HNSCC patients.

## INTRODUCTION

Head and neck cancer (HNC) is a heterogeneous disease that can involve multiple sites including oral cavity, nasopharynx, oropharynx, hypopharynx, larynx, paranasal sinuses and salivary glands. Head and neck squamous cell carcinoma (HNSCC) is the most common type, accounting for 90% of all HNC and ranking the sixth leading cancer by incidence worldwide [1]. Heavy use of tobacco and alcohol, human papillomavirus (HPV) infection and xenobiotic exposure are important risk factors for HNSCC [2, 3]. This disease is highly curable with a 5-year survival rate approximately from 40% to 60% [4]. The survival rate could be up to 80% for HNSCC patients with a disease at early stage [5, 6]. Metastasis and relapse, however, remain a clinical challenge in the management of HNSCCs.

Epithelial-mesenchymal transition (EMT) is a well-established critical mechanism for carcinoma metastasis. During this reprogramming process, epithelial cells acquire sufficient plasticity to become more motile and invasive [7, 8]. Several transcription factors (EMT-TFs) have been demonstrated critically involved in EMT regulation and TWIST is known as the principal inducer [9]. TWIST prompts EMT by repressing the expression of epithelial markers, such as E-cadherin, resulting in a loss of epithelial cell-cell adhesion and by upregulating the expressions of mesenchymal genes, such as N-Cadherin [10]. High expression of TWIST has been associated with aggressive tumor properties and poor survival in many cancers, including HNSCC, esophageal, and cervical SCC [11–13]. In mammals, there are two forms of TWIST, TWIST1 and TWIST2. The two isoforms share more than 90% sequence homology and structural similarity, have similar biochemical properties *in vitro*, and are co-expressed in many cell lines and tissues [14, 15]. However, the two isoforms can function differentially in carcinoma metastasis. For example, TWIST2 was reported to inhibit tumor formation in certain types of cancer [16–19]. Therefore, it is necessary to identify the prognosis roles of each isoform specifically.

Heavy tobacco use is an important risk factor for HNSCC [2, 3]. Cigarette smoking has been shown to promote EMT via upregulating EMT-associated gene expressions including *TWIST* [20]. However, whether smoking is associated with poor prognosis of HNSCC is controversial. Some reports argue that smoking [21] has no significant effect on HNSCC survival. In contrast, some studies suggest smoking may result in a significantly increased death risk in HNSCC [22, 23].

Gender may be another risk factor for HNSCC. The newly diagnosed cases in men are nearly 3-folds of those in women [24, 25]. In addition, the mortality of HNSCC in men is almost 2-folds higher than in women [24, 25]. However, a couple of studies did not found statistical difference between males and females in overall survival of HNSCC [26, 27]. Therefore, the better insight concerning risk factors for HNSCC is clearly needed.

Given that a high mortality rate is observed in male HNSCC and that tobacco use can regulate EMT, we asked whether gender and smoking could modify the effect of *TWIST1/2* on patient survival in HNSCC. Therefore, the aim of this study is to evaluate the association between *TWIST1*, *TWIST2* and both the clinicopathologic characteristics and survival of HNSCC patients, and the interplay between *TWIST* and either gender or smoking in the survival outcome.

## RESULTS

### Clinicopathologic characteristics of patients

Table 1 shows the clinical and pathologic characteristics of the 522 patients in the study. The average age of the patients was 60.9 years old (range from 19-90). The majority (73.7%, n=384) of patients were men, and 137 out of 521 were women. Of the patients, 88.1% were Caucasian, followed by African American (9.3%), Asian (2.2%) and American Native (0.4%). Among all the patients, there were 117 non-smokers and 391 smokers. More than half of the patients (n=284) had a stage IV disease, 105 stage III, 98 stage II and 20 stage I. There were 12.2% patients having a tumor with grade I, 58.8% with grade II and 29% with grade III. Tumors were mainly located at the tongue (n=200) and pharynx (n=135). In the patients, 490 were at M0 of metastasis stage and 26 at M1-MX stage. For the 183 patients who had post-radiotherapy information available, 121 (66.1%) received radiotherapy after surgery, while 62 did not. The average overall survival was 21.2 months with the range from 0.07 to 210.8 months for 519 patients with available follow-up information. During the follow-up, 222 of 521 patients with outcome information available died, 229 still lived at the end of the study, and the follow-up for the rest lost (or censored).

**Table 1:** Characteristics of patients and gene expressions of *TWIST1* and *TWIST2*

Variable	N	%	Range
Gender	521		
Female	137	26.3	
Male	384	73.7	
Race	506		
Caucasian	446	88.1	
African American	47	9.3	
Asian	11	2.2	
American Native	2	0.4	
Smoking	508		
No	117	23.0	
Yes	391	77.0	
Disease Stage	507		
I	20	3.9	
II	98	19.3	
III	105	20.7	
IV	284	56.1	
Tumor Grade	517		
I	63	12.2	
II	304	58.8	
III	150	29.0	
Tumor site	521		
Tongue	200	38.4	
Pharynx	135	25.9	
other	186	35.7	
Metastasis Stage	516		
M0	490	95.0	
M1-MX	26	5.0	
Radiotherapy	183		
No	62	33.9	
Yes	121	66.1	
Death	451		
No	229	57.4	
Yes	222	42.6	
Age (Mean ± SD*, years)	520	60.9 ± 11.9	19 - 90
Overall survival (months)	519	21.2	0.07 - 210.8
*TWIST1* (Median)	522	168.2	4.1 - 6812
*TWIST2* (Median)	522	55	0.9 - 407

* SD: standard deviation.

### TWIST1 and TWIST2 expression and their associations with clinicopathologic features in patients

The average expressions of *TWIST1* and *TWIST2* were 168.2 (range: 4.1 -6812) and 55 (range: 0.9 – 407), respectively, in these patients (Table 1). Associations of *TWIST1* and *TWIST2* were analyzed and the results are shown in Table 2. *TWIST1* and *TWIST2* levels in female and male patients were almost identical (p=0.846 for *TWIST1* and p=0.856 for *TWIST2*). No significant differences in *TWIST1* or *TWIST2* were found between Caucasian and other. Smokers (including ever and current smokers), however, had significantly higher levels of *TWIST2* than non-smokers (p = 0.015); while TWIST1 is not found different between the two groups. In addition, *TWIST1* and *TWIST2* levels were not significantly related to the stage of HDNC (I-IV). However,*TWIST2* levels were significantly associated with tumor grade (p <0.0001) with the lowest levels in Grade III. Neither tumor location nor metastasis stage was associated with *TWIST1* or *TWIST2* expressions. Radiotherapy did not change *TWIST1* or *TWIST2* expression.

**Table 2:** Associations of *TWIST1* and *TWIST2* expressions with clinicopathologic variables

Variable	N	*TWIST1*		*TWIST2*	
		Mean (SD)^1^	p value	Mean (SD)	p value
Gender	521		0.846		0.856
Female	137	2.20 (0.33)		1.70 (0.37)	
Male	384	2.19 (0.39)		1.69 (0.42)	
Race	506		0.994		0.688
Caucasian	446	2.19 (0.37)		1.69 (0.41)	
Other	60	2.19 (0.42)		1.71 (0.36)	
Smoking	508		0.377		0.015
No	117	2.22 (0.34)		1.62 (0.44)	
Yes	391	2.18 (0.38)		1.72 (0.39)	
Disease Stage	507		0.417		0.266
I	20	2.13 (0.28)		1.85 (0.36)	
II	98	2.16 (0.41)		1.72 (0.39)	
III	105	2.16 (0.39)		1.69 (0.42)	
IV	284	2.22 (0.37)		1.68 (0.40)	
Tumor Grade	517		0.275		<0.0001
I	63	2.12 (0.32)		1.72 (0.36)	
II	304	2.20 (0.37)		1.76 (0.37)	
III	150	2.21 (0.42)		1.57 (0.45)	
Tumor site	521		0.156		0.107
Tongue	200	2.19 (0.37)		1.65(0.43)	
Pharynx	135	2.14 (0.41)		1.74(0.36)	
other	186	2.22 (0.35)		1.71(0.40)	
Metastasis Stage	516		0.693		0.348
M0	490	2.19 (0.37)		1.70 (0.40)	
M1-MX	26	2.16 (0.52)		1.62 (0.43)	
Radiotherapy	183		0.118		0.304
No	62	2.15 (0.41)		1.73(0.40)	
Yes	121	2.25 (0.41)		1.67(0.38)	

^1^Mean(SD): mean (standard deviation) in log10.

### Associations of TWIST1 and TWIST2 with patient survival

**Figure 1:**
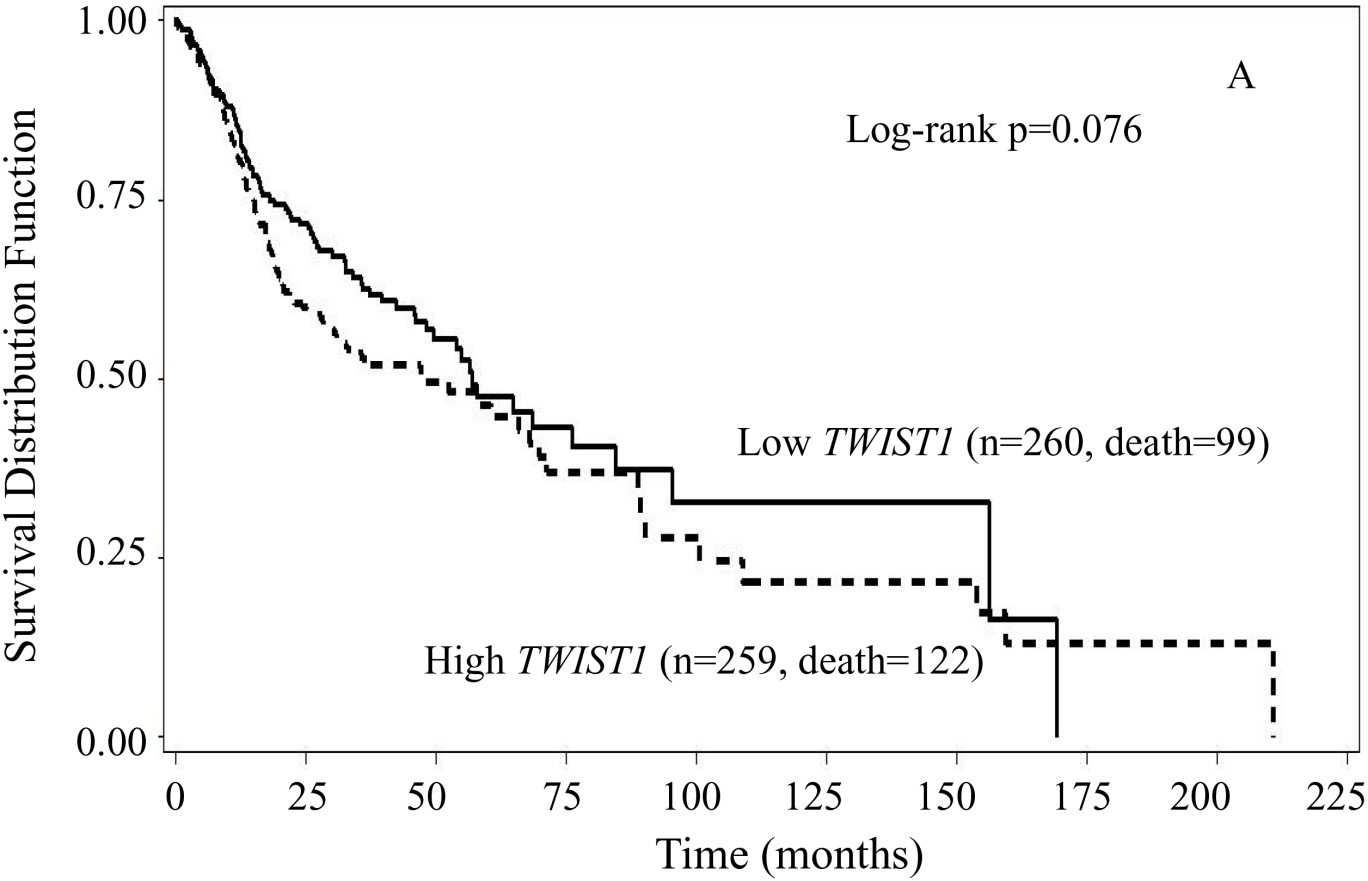

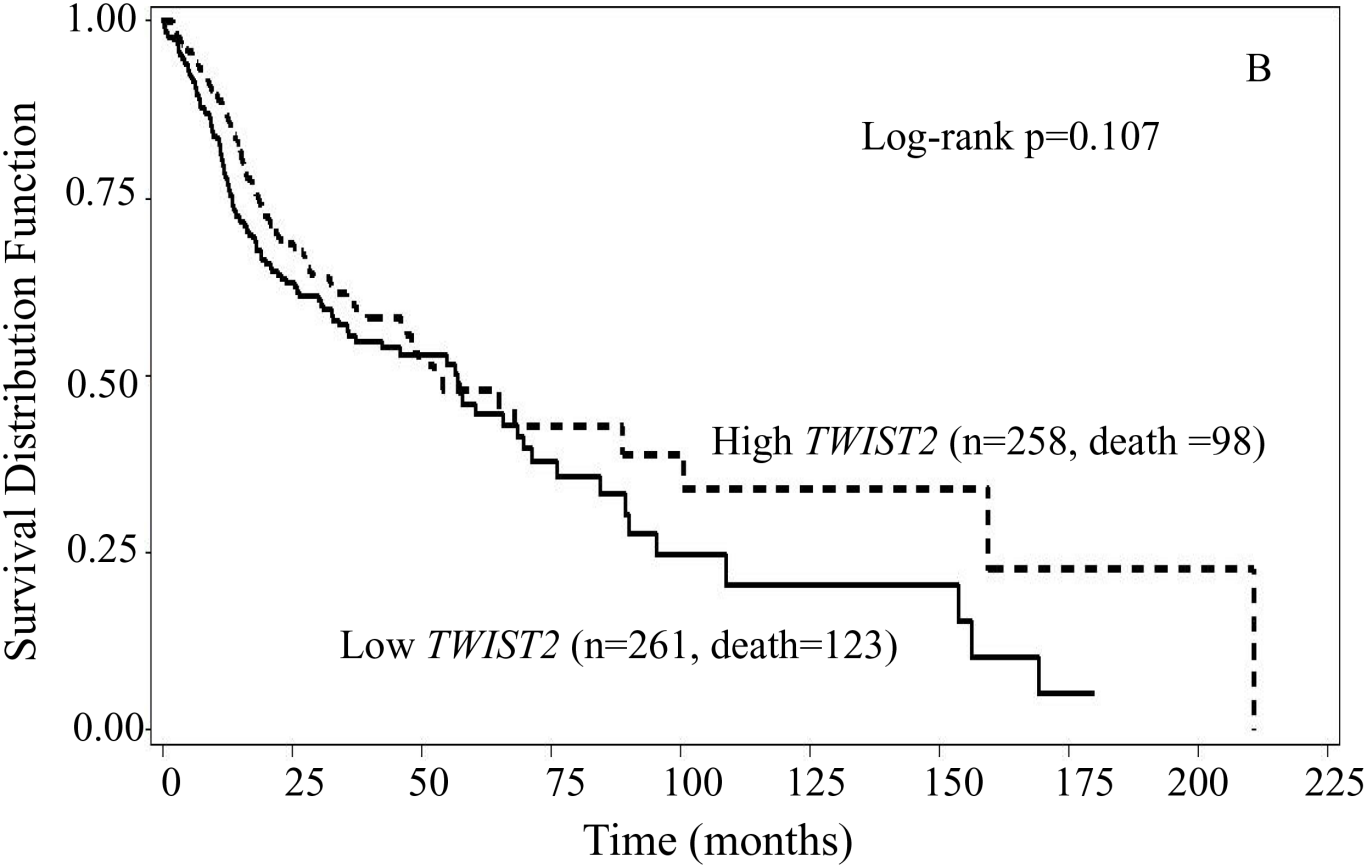
Kaplan-Meier overall survival curves stratified by either *TWIST1* (A) or *TWIST2* (B) expression levels In comparison to low expression, High *TWIST1* expression had inferior overall survival (log-rank p = 0.076), while high *TWIST2* had slightly but not statistically significant superior but overall survival at the beginning and after 60 months of follow-up (log-rank p =0.107).

To examine the associations of *TWIST1* and *TWIST2* gene expression with overall survival, we first performed Kaplan-Meier survival curves analyses stratified by either *TWIST1* or *TWIST2* expression levels. The results showed that patients with high *TWIST1* levels had inferior overall survival than those with low *TWIST1* (log-rank p = 0.076) (Figure 1A). The medians overall survival were 47.9 months (95% CI: 28.0 – 67.8 months) for those with high *TWIST1* and 56.9 months (95% CI: 48.2 – 84.4 months) for those with low *TWIST1*, respectively. The medians of overall survival were 52.3 months (95% CI: 45.9 – 100.5 months) for those with high *TWIST2* and 56.9 months (95% CI: 35.5 – 69.7 months) for those with low *TWIST2*, respectively. However, the survival curves were markedly separated with the superior overall survival for patients with high *TWIST2* after approximately 60 months compared to those with low. No statistically significant association was found between *TWIST2* expression and overall survival (Figure 1B, log-rank p = 0.107).

**Figure 2:**
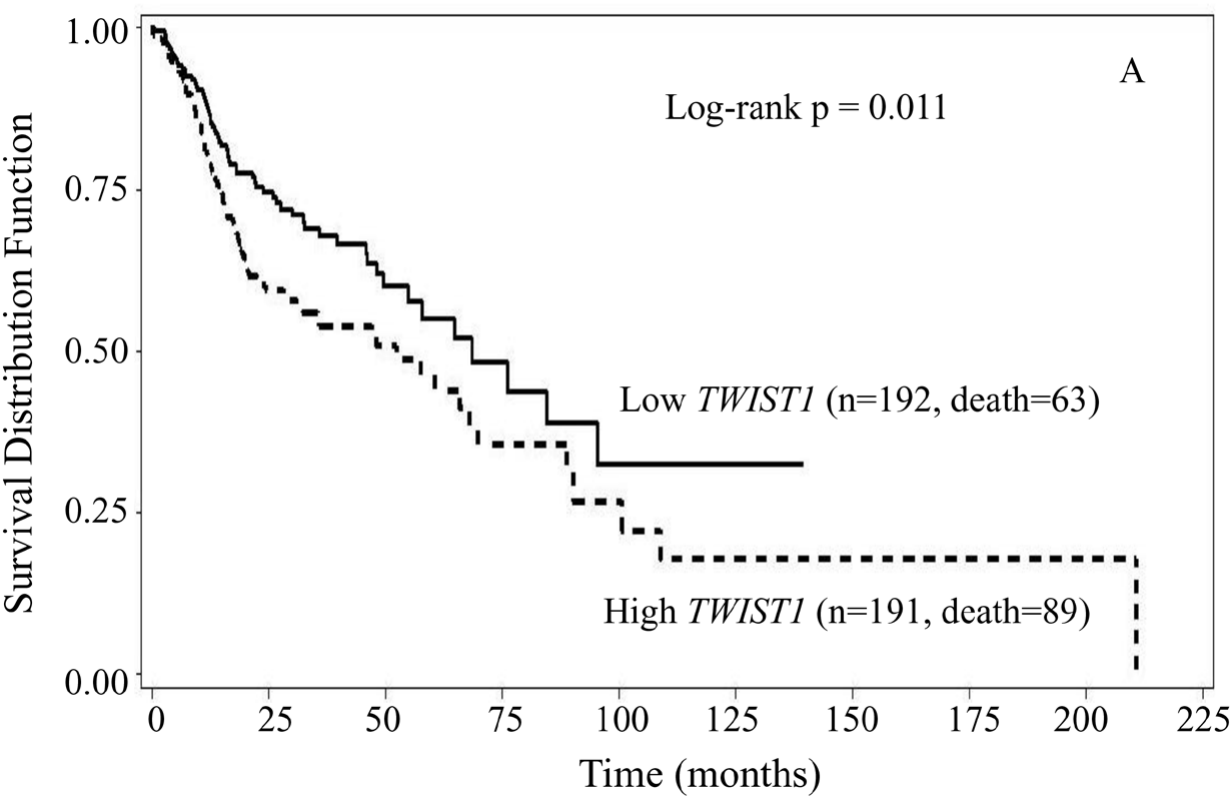

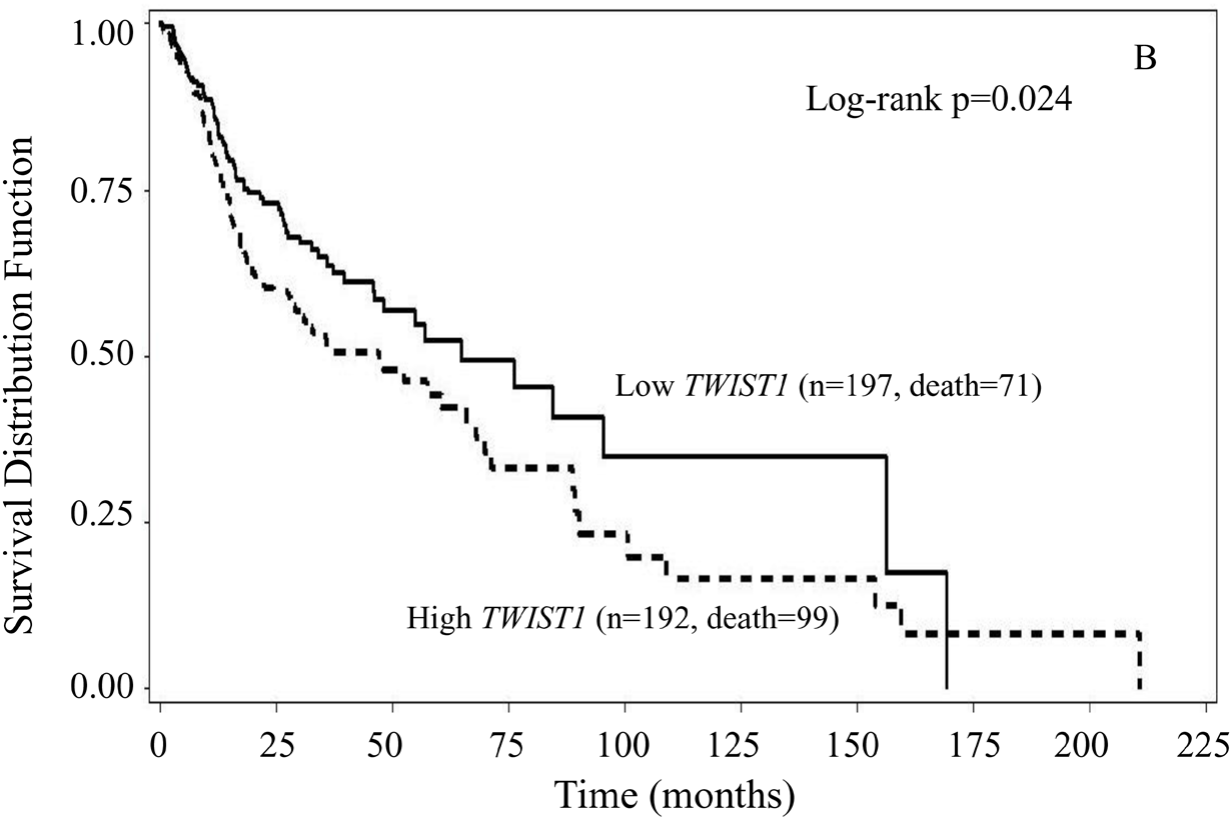

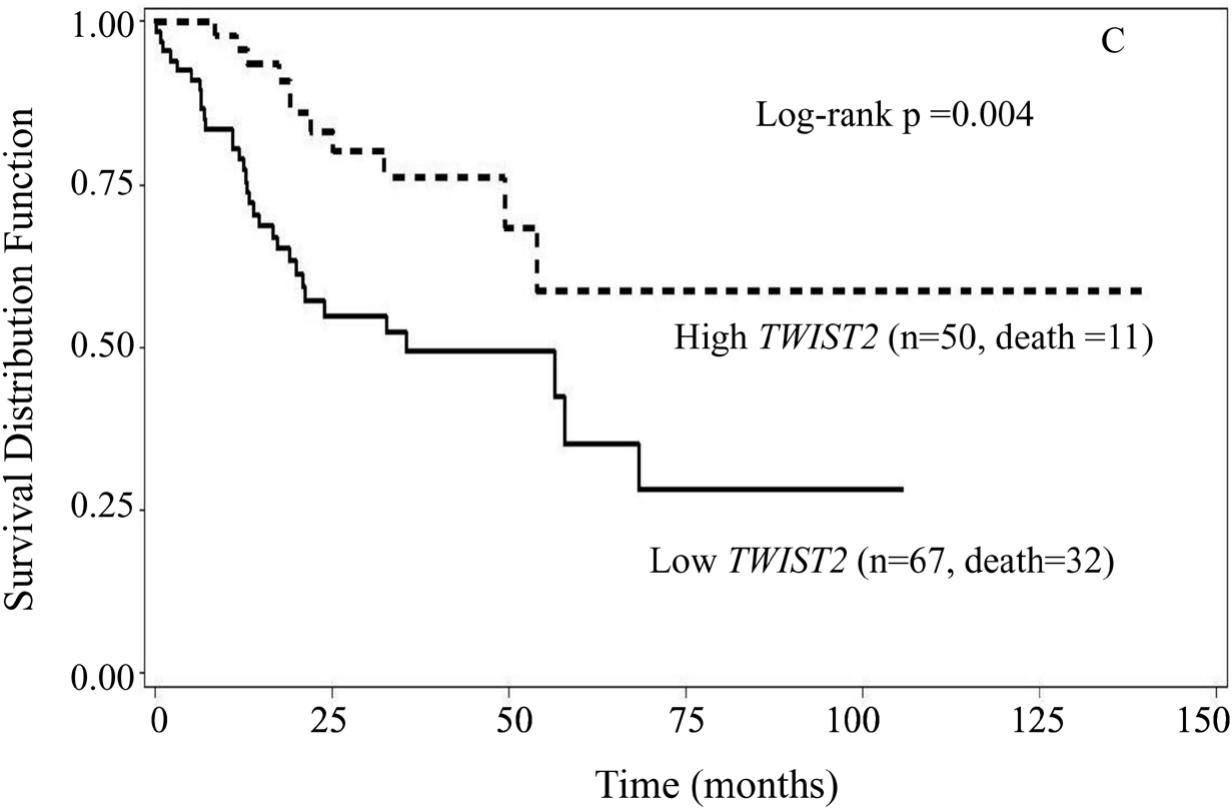
Kaplan-Meier overall survival curves stratified by either *TWIST1* or *TWIST2* in the subgroup of gender and smoking status High *TWIST1* had significantly inferior overall survival than low *TWIST1* in male patients **(A)** (log-rank p = 0.011), and smoker patients **(B)** (log-rank p = 0.024) compared to low *TWIST1*, respectively. In nonsmoker patients, high *TWIST2* had significantly superior overall survival in comparison to low one **(C)** (log-rank p = 0.004).

Since gender and smoking status have effects on HNSCC [24, 25], we then checked the associations of *TWIST1* and *TWIST2* expression levels with overall survival in subgroups of either gender or smoking status. We found that in the subgroups of males and smokers, patients with high *TWIST1* had worse survival than those with low *TWIST1*. In male patients with high *TWIST1*, the median overall survival was 52.3 months (95% CI: 28.3 – 67.8 months), whereas in male patients with low *TWIST1*, the median survival was 68.4 months (95% CI: 54.9 – 95.3 months) (Figure 2A, log-rank p = 0.011). In smoker patients with high *TWIST1*, the median overall survival was 47.0 months (95% CI: 28.3 – 65.8 months), whereas in smoker patients with low *TWIST1*, the median survival was 64.8 months (95% CI: 45.9 – 156.4 months) (Figure 2B, log-rank p = 0.024). However, in the subgroups of either female or non-smokers, no statistically significant association was found between *TWIST1* expression and overall survival (data not shown). In the subgroup of nonsmoker patients, those with low *TWIST2* expression had inferior overall survival, compared with those with high *TWIST2* (log rank p = 0.004) (Figure 2C); the survival rate did not reach 0.5 for the group with high *TWIST2*, and the median of overall survival was ∞ (95% CI: 49.4 - ∞ months) for high *TWIST2*. In contrast, the median survival time for the group with low *TWIST2* was 35.5 months (95% CI: 19.0 – 68.4 months). However, no statistically significant associations were found between *TWIST2* and overall survival in the subgroups of smoker patients, or male or female patients (data not shown).

We further performed multivariate Cox proportional hazard models to validate the Kaplan-Meier survival curves analyses with the adjustment of potential confounding variables and stratified by either gender or smoking status. Similarly, the multivariate Cox analysis showed that high *TWIST1* increased the risk of death, while high *TWIST2* decreased the risk of death (Table 3). The HRs were 1.37 (95% CI: 1.02 – 1.84) for high *TWIST1* in comparison to low one (p = 0.038), and 0.74 (95% CI: 0.55 – 1.01) for high *TWIST2* compared to low one (p = 0.055) with the adjustment of patients’ age at diagnosis, disease stage, tumor grade, tumor site, gender and smoking status. When patients were stratified by either gender or smoking status, those with high *TWIST1* had significantly elevated risk of death compared to those with low *TWIST1* in male (HR = 1.59, 95% CI: 1.12 – 2.25) (p = 0.010) and borderline significant elevated risk in smoker (HR = 1.38, 95% CI: 0.99 – 1.94) (p =0.058) subgroups (Table 4) after adjusting covariates. In nonsmokers, patients with high *TWIST2* had significantly decreased risk of death compared to those with low one; the adjusted HR was 0.38 (95% CI: 0.18 – 0.80) (p = 0.011). However, when we stratified the patients with either *TWIST1* or *TWIST2* levels, we observed that there were no significant associations between either gender or smoking status and the risk of death in the multivariate Cox proportional hazard analyses (data not shown).

**Table 3:** Associations of *TWIST1* and *TWIST2* expressions with the risk of death

Variable	HR^1^	95% CI^2^	p value
TWIST1 (high vs low)	1.37	1.02 - 1.84	0.038
TWIST2 (high vs low)	0.74	0.55 – 1.01	0.055
Smoking (yes vs no)	1.17	0.81 - 1.68	0.403
Gender (male vs female)	0.87	0.63 - 1.20	0.406
Age (per 5 years)	1.10	1.02 - 1.18	0.010
Stage	1.06	0.90 - 1.25	0.459
Grade	1.02	0.82 - 1.26	0.886
Tumor site			
Tongue	1.00		
Pharynx	0.97	0.65 – 1.44	0.865
Other	1.18	0.84 – 1.67	0.345

^1^HR, hazard ratio, which was estimated in multivariate Cox proportional hazard regression analysis.

^2^CI: confidence interval.

**Table 4:** Associations of *TWIST1* and *TWIST2* expressions with the risk of death stratified by either gender or smoking status

Stratefied variabel	Variable	adj-HR^1^	95% CI^2^	p value
Gender^3^				
Female	*TWIST1* (high vs low)	1.07	0.61 - 1.88	0.802
	*TWIST2* (high vs low	0.68	0.38 - 1.20	0.181
Male	*TWIST1* (high vs low)	1.59	1.12 - 2.25	0.010
	*TWIST2* (high vs low)	0.74	0.51 - 1.05	0.094
Smoking status^4^				
No	*TWIST1* (high vs low)	0.67	0.32 - 1.44	0.309
	*TWIST2* (high vs low	0.38	0.18 - 0.80	0.011
Yes	*TWIST1* (high vs low)	1.38	0.99 - 1.94	0.058
	*TWIST2* (high vs low)	0.90	0.64 - 1.27	0.559

^1^adj-HR: adjusted hazard ratio, which was estimated in multivariate Cox proportional hazard regression analyses.

^2^CI: confidence interval.

^3^covariates included patient age (per 5 years), disease stage, tumor grade, smoking status and tumor site.

^4^covariates included patient age (per 5 years), disease stage, tumor grade, gender, and tumor site.

## DISCUSSION

In this study, we demonstrated the associations of the two isoforms of a critical EMT regulators, *TWIST1* and *TWIST2*, with clinicopathological characteristics and overall survival in HNSCC and their interactions with either gender or smoking in the risk of death using a publicly accessed TCGA Head and Neck squamous cell carcinoma dataset. We found that *TWIST1*, but not *TWIST2*, was significantly positively linked to overall survival of these patients. Further analysis stratified with gender and tobacco use revealed this significant correlation only existing in males or smokers but not significantly in female or non-smokers. Unexpectedly, although *TWIST2* levels were not significantly related with overall survival, higher *TWIST2* levels were correlated with lower grade in overall patients and better survival in non-smokers; interestingly, this beneficial relation of *TWIST2* with survival is lost in smokers.

Generally, disease stage and age are important unfavorable prognostic marker in human cancer. Unexpectedly, disease stage was not significantly associated with the risk of death in this study. However, we did find that patients’ age at diagnosis increased the mortality of HNSCC. These results suggest that the finding might not result from by chance. In addition, we could not rule out the possibility that the sample size in early stage (stage I: 20/507; stage II: 98/507) was relatively small, while the majority of patients were diagnosed at advanced stage in this study, which might result in insufficient power to detect an association between disease stage and risk of death. Furthermore, more than 25% of stage I and II oral cavity cancer patients can develop recurrence, a culprit of death [28].

To our knowledge, this is the first study to evaluate the respective role of *TWIST1* and *TWIST2* in HNSCC with a relatively large sample size. Unlike many studies which include patients who were followed up to 36 or 70 months, as long as up to 210.8 months were followed up for the patients.

Since TWISTs were reported as the master regulator in tumor metastasis in 2004 [29], multiple studies have demonstrated TWIST as a potential prognostic marker for many cancers, including myeloid leukemia, oral, esophageal, lung, breast, cervical and bladder cancer [27, 30–34]. TWIST enhances carcinoma metastasis by promoting cell proliferation, migration, invasion and colony formation. High TWIST protein expression has been shown in a positive correlation with poor prognosis in many cancers, including HNSCC [7, 26, 27, 35–37]. TWIST1 and TWIST2 share more than 90% sequence homology and structural similarity, and they have similar biochemical properties *in vitro*, and overlap in localization and expression pattern [14, 15, 38]. Thus, it was thought that overexpression of TWIST1 and/or TWIST2 was similarly correlated to poor prognosis in HNSCCs [36, 37]. In several meta-analysis studies, TWIST1 and TWIST2 were treated as identical and the data were combined together [27, 39]. However, there is evidence indicating that the two isoforms function differentially in carcinoma metastasis [40]. For example, the prognostic value of TWIST1 has been consistently demonstrated in many cancers; while although TWIST2 was also a prognostic marker in certain types of cancer, it was not associated with invasion and metastasis in hepatocellular carcinoma [16]. Moreover, TWIST2 was reported to inhibit tumor formation in a mouse osteosarcoma model [17]. In acute lymphoblastic leukemia, overexpression of TWIST2 inhibits cell growth, prompts apoptosis and increases sensitivity to chemotherapeutic agents [18]. TWIST2 may act as a tumor repressor by activating known tumor-suppressor genes [17, 19]. Indeed, the prognostic value of *TWIST* in HNSCC is still controversial or even opposite. Some paper reported the association of TWIST1 with poor prognosis in ESCC patients [12, 41, 42]; whereas there are also studies showing that TWIST is not associated with EMT of esophageal adenocarcinoma [43–45]. In our study, we found that higher *TWIST1* was associated with poor survival and this relationship was more noticeable in smokers or males; unexpectedly, higher *TWIST2* indicates a better survival in non-smokers. Based on the findings in this study, *TWIST1* showed a risk factor increasing the mortality in HNSCC, particularly in male patients, whereas *TWIST2* exhibited a protective role in decreasing the mortality in HNSCC, particularly in non-smoker patients.

In our study, we analyzed whether gender and smoking could modify the effect of *TWIST1/2* levels on the risk of death in HNSCC patients. In consistent with other studies [26, 27], our analysis did not find an impact of either gender or smoking on the risk of death. In addition, TWIST1 expression was not associated with gender in this study, which is in the agreement with the other previous results that TWIST expression was not related to gender in nasopharyngeal cancer [26]. However, we found that higher TWIST1 was linked to poor survival only in males but not in females. Taken together, our study firstly suggests that gender might be a modifier rather than a confounder in the effect of TWIST1 expression on patient survival in HNSCC.

Smoking is a known primary risk factors for HNSCC [46, 47], and tobacco use is reported in correlation with higher TWIST expression. Benzo(a)pyrene (BaP), one of the major chemical substance in cigarette, was found to enhances cell migration and invasion as well as TWIST expression in non-small cell lung cancer (NSCLC) cell line A549. In addition, the knockdown of TWIST blocked the migration and invasion of A549 cells induced by BaP [48], implying that smoking may cause EMT through TWIST. Our study revealed that the mRNA levels of *TWIST1* were not affected by smoking. This is in consistent with study from the other lab [49]. However, the inverse relationship of *TWIST1* with survival becomes even more noticeable in smokers. Interestingly, although *TWIST2* level is slightly higher in smokers than in non-smokers, the beneficial role of *TWIST2* on survival rate is still lost in smokers. These data suggest that smoking may modify the effect of *TWIST2* expression on the patient survival in HNSCC.

In summary, this study revealed that *TWIST1* and *TWIST2* were differently involved in prognostic of HNSCC with high *TWIST1* linking to poor survival, which is more obvious in either males or smokers than in either female or non-smokers. In addition, high *TWIST2* seems to decrease the risk of death in non-smoker but not in smoker patients. These findings suggest *TWIST1* and *TWIST2* are potential prognostic markers of HNSCC, and gender and smoking status can modify the effect of *TWIST1* and *TWIST2* expression on the risk of death in HNSCC.

## MATERIALS AND METHODS

### Gene expression and clinicopathologic data

We retrieved upper quartile normalized RNA-seq V2 RSEM values of *TWIST1* and *TWIST2* expressions from a TCGA Head and Neck squamous cell carcinoma dataset, which is available at TCGA provisional (http://www.cbioportal.org/). Experimental data generation and processing were conducted as previously described [50]. After downloading clinicopathologic data on these patients, we assembled gene expressions and clinicopathologic data for data analyses.

### Statistical analyses

Statistical analyses were performed using SAS version 9.2 (SAS Institute, inc). The overall survival in months was calculated as the time from surgery until the occurrence of death. General linear models (GLM) were used to analyze the associations of *TWIST1* and *TWIST2* with clinicopathologic features and general characteristics of patients, in which the expressions of *TWIST1* and *TWIST2* were further converted by logarithm. The logarithm conversion was made after one plus each of the values to avoid impossible conversion for those with a value of zero. The median of either *TWIST1* or *TWIST2* expression levels was used as a cutoff value in classifying patients into two groups, low or high, in survival analyses. Kaplan-Meier survival curves were used to assess the associations of *TWIST* genes with patient survival, and multivariate Cox proportional hazards regression models were used to estimate the hazards ratios (HRs) and their 95% confidence intervals (95% CIs) with the adjustment for patients’ age at diagnosis, disease stage, tumor grade, gender, smoking status and tumor site. A p value of less than 0.05 was considered statistically significant.
